# Sustained low catheter related infection (CRI) incidence in an observational follow-up study of 9924 catheters using automated data scripts as quality assurance for central venous catheter (CVC) management

**DOI:** 10.1016/j.infpip.2023.100273

**Published:** 2023-02-19

**Authors:** Mika M. Rockholt, Tobis Agrell, Hulda Thorarinsdottir, Thomas Kander

**Affiliations:** a Department of Intensive and Perioperative Care, Skåne University Hospital, Lund, Sweden; b Department of Anesthesiology, Perioperative Care and Pain Medicine, NYU Langone Health, NYC, NY, USA; c Department of Clinical Sciences, Lund University, Sweden; d Department of Intensive and Perioperative Care, Landspitali University Hospital, Reykjavik, Iceland

**Keywords:** Catheter-related infections, Central venous catheter management, Healthcare quality assurance, Follow-up study, Automatic data extraction, Simple queries

## Abstract

**Background:**

To maintain a low incidence of Catheter Related Infections (CRI) and Catheter Related Bloodstream Infections (CRBSI), continuous follow-up studies on catheter management are necessary. The aims of the present study were to investigate the incidence of catheter tip colonisation, CRI and CRBSI in the Region, to further explore the feasibility of automatic data collection and to investigate associations between independent variables and CRI.

**Methods:**

Data from electronic patient charts on all documented central venous catheter (CVC) insertions from multiple hospitals in southern Sweden, between March 2019 and August 2020, were automatically extracted. Multivariable regression analyses were used to identify associated risk factors.

**Results:**

In total, 9924 CVC insertions were included. The prevalence of CRI and CRBSI were 0.7% (*n = 74*) and 0.02% (*n = 20*) with incidences of 1.2/1000 catheter days and 0.3/1000 catheter days, respectively.

**Conclusions:**

We found a sustained low incidence of CRI and CRBSI in the Region. Catheter tips were less likely to be colonised when the subclavian route was used compared to the internal jugular route and male sex as well as increased number of catheter lumens were associated with both catheter tip colonisation and CRI. By using automated scripts, data extraction was efficient and feasible but also demonstrated that real-time quality assurance should be recommended, since this is superior to current standard.

## Introduction

Over the last decades, improvements in the management of central venous catheters (CVCs) have been made to reduce catheter related infections (CRIs), including catheter related bloodstream infections (CRBSIs). However, CRI continues to be a problem, causing not only increased patient suffering but also a burden on healthcare economy with reports showing that one CRI can cost up to $32000 [1].

The view on CVC management and CRI prevention shifted in 2006 when Pronovost *et al.* described simple evidence-based interventions resulting in significant CRI reduction [2,3]. These interventions, also known as “insertion bundles” or “hygiene bundles”, were implemented worldwide resulting in multiple reports on lowered CRI incidences [3]. In line with Pronovost's study, we introduced a simple hygiene insertion bundle in 2012 which was demonstrated effective as it reduced the incidence of CRI [4].

In contrast to the numerous publications reporting short-term low incidences of CRI after the introduction of various hygiene bundles [2,4,5], there are relatively few follow-up studies evaluating CRI incidences over time and focusing on the long-term effects of hygiene bundles. In the past decade, one quality improvement report published in 2011 by Batistella *et al.* [6] and one follow-up study from 2014 by Hammarskjöld *et al.* [7] both describe a sustained low catheter infection rate, six years after implementing new catheter insertion routines. In summary, these studies confirmed safe and effective implementation of new CVC-management strategies. However, more recent reports on CRI incidences with follow-up studies worldwide are scarce.

In parallel, modernisation of health records and patient data over the last decades has led to the development of electronic health record (EHR) systems comprised by valuable data used in epidemiological studies, such as the ones reporting catheter-related and other life-threatening infections [8,9]. Several recent studies have reported electronic surveillance systems and the use of data engines and search queries to monitor and report the management of CVCs, allowing tracking of compliance to CVC hygiene insertion bundles but also to monitor the infection and complication frequencies [8,10,11].

In an attempt to evaluate long-term effects of the hygiene insertion bundle introduced in 2012 at the current institution, we used automated script-based search in the EHR to conduct this multicenter observational follow-up study as a quality assurance measure of CRI over time. The primary aim of this study was to investigate the incidence of CRI and CRBSI using an automated script-based method in an unselected, large, cohort of patients with central venous access [4]. Secondarily, we aimed to identify associated risk factors for CRI.

## Methods

This study was approved by the Swedish Ethical Review Authority (dnr 2014/916 and 2018/866) and requirement for written informed consent was waived. The study was carried out at the Department of Intensive and Perioperative Care at Skåne University Hospital, Lund, Sweden. The manuscript was prepared according to the STROBE guidelines for observational studies.

All documented CVC insertions from ten different hospitals within the Scania Region (Region Skåne), Sweden, from March 2019 to August 2020 were eligible for inclusion. Exclusion criteria included patients under 8 years of age, missing insertion date or unknown insertion site. Peripherally inserted catheters (PICC-lines) and subcutaneous venous ports were not included as they were inserted using different techniques and different hygiene precautions.

### CVC insertion, management, and removal

CVC insertions were performed according to Regional guidelines, previously described^(4, 12)^. Some of the variables included in the template are described in Table I*.*

**Table I:** Baseline variables for patients receiving a central venous catheter (CVC)^a^

	Jugular vein n=8398	Subclavian vein n=1330	Femoral vein n=196	All n=9924
Number of patients	5989	1176	169	6872
Age, years	70 [58–77]	67 [53–75]	60 [46–73]	69 [57–76]
Sex, male	5099 (61)	828 (62)	111 (57)	6038 (61)
**Indication for CVC**^b^				
Vessel irritating medication	2797 (33)	583 (44)	72 (37)	3452 (35)
Cardiac surgery	2820 (34)	277 (21)	9 (5.0)	3106 (31)
Parenteral nutrition	1251 (15)	196 (15)	9 (5.0)	1456 (15)
Haemodynamic monitoring	2007 (24)	259 (19)	30 (15)	2296 (23)
Peripheral venous access impossible	1640 (20)	339 (25)	34 (17)	2013 (20)
Blood sampling	2436 (29)	461 (35)	45 (23)	2942 (30)
Fluid resuscitation	1496 (18)	143 (11)	43 (22)	1682 (17)
Others	1176 (14)	101 (8.0)	86 (44)	1363 (14)
Missing	174 (2.0)	58 (4.0)	6 (3.0)	238 (2.0)
**Type of catheter**				
Central Venous Catheter (CVC)	8036 (96)	1312 (99)	142 (72)	9490 (96)
Central Haemodialysis Catheter (CHC)	362 (4.0)	18 (1.0)	54 (28)	434 (4.0)
**Number of CVC lumen**				
1	2275 (27)	353 (27)	19 (9.0)	2647 (27)
2	1859 (22)	311 (23)	19 (9.0)	2189 (22)
3	2374 (28)	380 (29)	41 (22)	2795 (28)
4	587 (7.0)	74 (5.0)	5 (3.0)	666 (7.0)
5	799 (10)	158 (12)	46 (23)	1003 (10)
Missing	504 (6.0)	54 (4.0)	66 (34)	624 (6.0)
**Anticoagulant treatment before insertion**^c^				
No	5441 (65)	952 (72)	128 (65)	6521 (66)
Warfarin	374 (4.0)	43 (3.0)	2 (1.0)	419 (4.0)
Non-vitamin K Antagonist Oral Anticoagulants	427 (5.0)	45 (3.0)	6 (3.0)	478 (5.0)
Acetylsalicylic acid	965 (11)	132 (10)	14 (7.0)	1111 (11)
Low Molecular Weight Heparin	709 (8.0)	89 (7.0)	18 (9.0)	816 (8.0)
Other	762 (9.0)	103 (8.0)	35 (18)	900 (9.0)
**Procoagulant treatment before insertion**^d^				
No	7282 (87)	1159 (87)	153 (78)	8594 (87)
Platelet transfusion	110 (1.0)	54 (4.0)	8 (4.0)	172 (2.0)
Activated prothrombin complex	217 (3.0)	18 (1.0)	9 (5.0)	244 (2.5)
Vitamin K	82 (1.0)	6 (0.5)	5 (3.0)	93 (1.0)
Fibrinogen	35 (0.4)	7 (0.5)	3 (2.0)	45 (0.5)
Plasma	49 (0.5)	7 (0.5)	3 (2.0)	59 (1.0)
Tranexamic acid	71 (0.8)	11 (1.0)	4 (2.0)	86 (1.0)
Desmopressin	15 (0.2)	3 (0.2)	1 (1.0)	19 (0.2)
Other	753 (9.0)	96 (7.0)	27 (14)	876 (9.0)
**Room for CVC-insertion**				
Operating theatre	4942 (59)	395 (30)	33 (17)	5370 (54)
Intensive Care Unit	2022 (24)	469 (35)	125 (64)	2616 (26)
Room reserved for CVC-insertion	902 (11)	399 (30)	22 (11)	1323 (13)
General ward	242 (3.0)	40 (3.0)	6 (3.0)	288 (3.0)
Missing	290 (3.0)	27 (2.0)	10 (5.0)	327 (3.0)
**Department of admission at CVC insertion**				
Surgical ward	4504 (54)	513 (39)	28 (14)	5045 (51)
Medical ward	2282 (27)	569 (43)	59 (30)	2910 (29)
Intensive Care Unit	1335 (16)	198 (15)	91 (46)	1624 (16)
Missing	277 (3.0)	50 (4.0)	18 (9.0)	345 (4.0)
**Number of skin punctures**				
1	6302 (75)	1017 (76)	149 (76)	7468 (75)
2	1333 (16)	193 (15)	20 (11)	1546 (16)
3	437 (5.0)	67 (5.0)	12 (6.0)	516 (5.0)
4	100 (1.0)	21 (2.0)	1 (0.5)	122 (1.0)
5 or more	45 (0.5)	12 (1.0)	3 (1.0)	60 (0.5)
Missing	181 (2.5)	20 (1.0)	11 (5.5)	212 (2.0)
**Number of vessel punctures**				
1	6584 (78)	1130 (85)	154 (79)	7868 (79)
2	1209 (14)	143 (11)	18 (8.5)	1370 (14)
3	334 (4.0)	22 (1.5)	5 (3.0)	361 (4.0)
4	51 (0.5)	7 (0.5)	1 (0.5)	59 (0.5)
5 or more	16 (0.5)	2 (0.5)	2 (1.0)	20 (0.5)
Missing	204 (3.0)	26 (1.5)	16 (8.0)	246 (2.0)
**Immediate mechanical complications**				
Any	453 (5.5)	66 (5.0)	27 (14)	546 (5.5)
Failed insertion^e^	177 (2.1)	27 (2.0)	12 (6.1)	216 (2.2)
Bleeding^f^	131 (1.6)	13 (1.0)	10 (5.1)	154 (1.6)
Punctured artery	87 (1.0)	16 (1.2)	5 (2.6)	108 (1.1)
Arrhythmia	47 (0.6)	6 (0.5)	0 (0.0)	53 (0.5)
Pneumothorax	11 (0.1)	4 (0.3)	0 (0.0)	15 (0.1)
**Total catheter days**	48899	12725	916	62540
Days with catheter	5 [2–9]	6 [3–14]	3 [1–7]	5 [2–10]
Missing	1779 (21)	260 (20)	31 (16)	2070 (21)

a Numbers are presented as number (%) and continuous variables are presented as median [interquartile range].

b Registering multiple indications for one insertion was possible. Example of indications labeled as “Other” were introducer, pacemaker, continuous renal replacement therapy or dialysis.

c Registering multiple anticoagulative treatments for one insertion was possible.

d Registering multiple thrombotic treatments for one insertion was possible.

e “Failed insertions” included insertions with change of blood vessel and insertion attempts where no CVC was inserted.

f Grade 1 bleedings were not registered in this study. In this study 99.4 % of all bleedings could be classified according to Common Terminology Criteria for Adverse Events (CTCAE; Version 5.0) as grade 2. One grade 4 bleeding occurred also included in the” Bleeding” category.

Catheter tips were only cultured if a CRI was suspected. CVCs were removed after site treatment with 0.5% chlorhexidine in 70% alcohol. The distal end of the CVC was submerged into a culture tube, and the distal 5cm was cut off. The tip was cultured using a semi-quantitative method where growth of >102 CFU/catheter tip was considered significant colonization [13,14]. Blood cultures taken between 0 - 48h after CVC removal were included in the data set. The automated BACT/ALERT®-system (BioMérieux, Marcy l'Etoile, France) was used and all cultures were incubated until microbial growth was detected or for a maximum of five days.

### Data extraction

By using an automated script-base search in the EHR (Melior, Cerner, North Kansas City (MO), USA), all documented CVC-insertion templated, during the study period, were extracted. Automatically extracted data was directly inserted into a compiled, encrypted database (Excel, version 10, Microsoft, Santa Rosa, USA), where each individual insertion was merged with matching microbiological data, laboratory values obtained within 48 hours prior to the CVC removal. All insertions with data fulfilling the exclusion criteria were removed from the database.

### Outcomes and definitions

The primary outcomes were defined according to the definitions used by Centers for Disease Control and Prevention (CDC) [15]. Catheter tip colonisation was defined as a positive tip culture in a patient where suspected catheter infection had led to removal of the CVC, regardless of clinical symptoms. CRI was defined as positive tip culture combined with two or more systemic inflammatory response syndrome (SIRS) criteria (fever >38 or <36 C^o^, respiratory rate >20 breaths per minute, heart rate >90 beats per minute or white blood cell count >12000/μL or <4000/μL) upon CVC removal and no likely explanation other than the catheter. The diagnosis of CRBSI required fulfillment of the CRI-criteria combined with a peripheral blood culture taken within 48h prior to CVC removal, with the same microorganism isolated in both cultures.

### Statistics and analyses

All analyses were performed using SPSS (version 28, IBM, New York, USA) using data from the original dataset. Results were expressed as a median [interquartile range] for continuous variables and a number (percentage) for categorical variables. Baseline variables were considered as potential independent variables and differences between cases with tip colonisation and CRI were tested against controls using univariate regression analyses. Given a presumed complex interdependence of the independent variables, we also performed a multivariable logistic regression for all outcomes. The number of independent variables in the multivariable logistic regression models was limited so that maximum one independent variable per ten events was included. The selection of independent variables in the multivariable regression model was based on results from previous studies and results from the univariate analyses [4,7,12,16]. The Hosmer-Lemeshow test was used to test goodness of fit for multivariable testing. *P*<0.05 was considered significant and all tests were two tailed.

## Results

**Figure 1:**
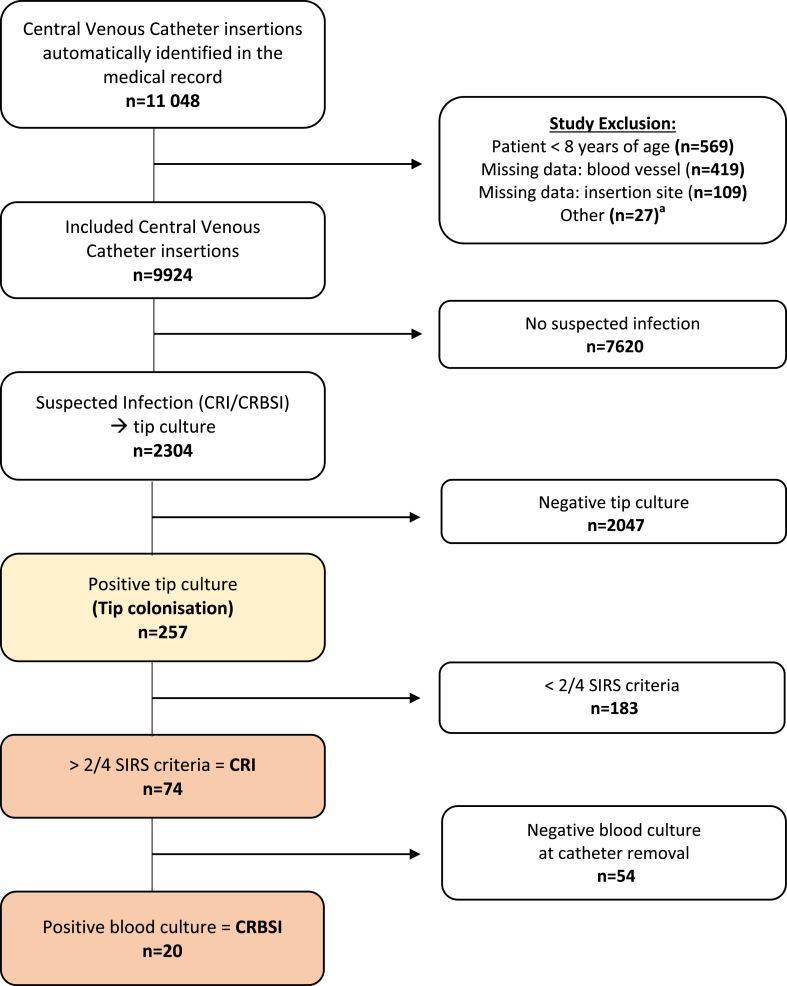
Flow chart with the number of Catheter Tip Colonisations, Catheter Related Infections (CRI) and, Catheter Related Bloodstream Infection (CRBSI) between March 2019 and August 2020 in Region Skåne, Sweden. Abbreviations: SIRS, systemic inflammation response syndrome.^**a**^ Peripherally inserted catheters, or insertion missing both insertion site and insertion date.

In summary, a total of 9924 catheter insertions in 6872 patients were included in the study during the study period of 18 months (Figure 1). Data from the CVC insertion template, automatically extracted from the EHR, are presented as baseline characteristics of patients and CVCs in Table I***.*** The most common insertion site was the jugular vein (85%) and the majority of catheters were inserted in an operating theatre (54%). Immediate complications after CVC insertion occurred in 5.5% of cases, where failed insertion (change of blood vessel or abandoned attempt of insertion) was most common (2.2%), followed by bleeding (1.6%) and punctured artery (1.1%).

In total, 2304 (23%) CVCs were sent for culture (Figure 1)*.* A total of 257 (2.6%) of all 9924 catheters demonstrated a positive tip culture, yielding a colonisation incidence of 4.1 tip colonisations/1000 catheter days. Further, CRI was confirmed in 74 cases (0.7%) yielding a CRI incidence of 1.2/1000 catheter days. Simultaneous blood culture and tip cultures were obtained in 667 cases (6.7%) with suspected infection where these cultures yielded positive results in 69 cases (0.7%). However, only 20 cases met the criteria for CRBSI (0.2%), resulting in a CRBSI incidence of 0.3/1000 catheter days. Due to unknown catheter durations, a total of 2070 catheters (all without colonisation and positive tip culture) were excluded when calculating the incidences.

To evaluate any impact on the COVID-19 pandemic, a comparison between the prevalence of catheter colonisation/CRI/CRBSI during six months in the pandemic and the corresponding six months the year before, was performed. The analyses demonstrated no differences between the periods (Table II).

**Table II:** Impact of COVID-19 pandemic on colonisation, CRI and CRBSI

Period	Colonisation (n=257)	CRI (n=74)	CRBSI (n=20)
March 2019–August 2019	94	27	7
March 2020–August 2020	96	28	7
	*P = 0.8538*	*P = 0.9652*	*P = 0.9283*

The study was conducted between March 2019 and August 2020. During this time-period, the included hospitals received patients with COVID-19 between March 2020 and August 2020. Prevalences were compared between March 2019–August 2019 and March 2020 -August 2020. Using the Chi-square test, no significant difference in infection prevalence was seen between the COVID-19 free period and the first wave of the COVID-19 pandemic.

All isolated organisms are presented in Table III***.*** The pathogens isolated in tip and blood cultures predominantly consisted of *Staphylococci*, where *Staphylococcus epidermidis* was by far the most common species. In fact, *S. epidermidis* was the only coagulase negative staphylococcus identified in CRI and CRBSI. In tip cultures *S. epidermidis* was seen in 64% and *S. aureus* in 12% of all tip cultures. The microorganisms responsible for CRI and CRBSI respectively was *S. epidermidis* (66% and 40%), *S. aureus* (15% and 35%), various Gram negatives (12% and 10%) and yeasts (12% and 10%)

**Table III:** Isolated microorganisms from central venous catheter (CVC) tips^a^

Organism	Colonised tips (n=257)	CRI (n=74)	CRBSI (n=20)
**Gram positives:**	216 (84)	66 (89)	16 (80)
*Staphylococcus*	164 (64)	49 (66)	8 (40)
Coagulase negative (total)^b^			
*S*. *epidermidis*	*152 (59)*	*49 (66)*	*8 (40)*
*S. warneri*	*1 (0.4)*	*0 (0.0)*	*0 (0.0)*
*S. capitis*	*5 (1.9)*	*0 (0.0)*	*0 (0.0)*
*S. caprae*	*2 (0.8)*	*0 (0.0)*	*0 (0.0)*
*S. hominis*	*1 (0.4)*	*0 (0.0)*	*0 (0.0)*
*S. haemolyticus*	*1 (0.4)*	*0 (0.0)*	*0 (0.0)*
*S. simulans*	*1 (0.4)*	*0 (0.0)*	*0 (0.0)*
*S. lugdunensis*	*1 (0.4)*	*0 (0.0)*	*0 (0.0)*
*Staphylococcus aureus*	*32 (12)*	*11 (15)*	7 (35)
other	*8 (3.0)*	*1 (1.0)*	0 (0.0)
*Enterococcus*			
*faecalis*	*3 (1.0)*	*1 (1.0)*	0 (0.0)
*faecium*	*4 (2.0)*	*3 (4.0)*	1 (5.0)
Other	*5 (2)*	*1 (1)*	0
**Gram negatives:**	**22 (9.0)**	**9 (12)**	**2 (10)**
*Pseudomonas aeruginosa*	*5 (2.0)*	*3 (4.0)*	1 (5.0)
*Klebsiella*			
*pneumoniae*	4 (2.0)	1 (1.0)	0 (0.0)
*aerogenes*	2 (0.8)	2 (3.0)	0 (0.0)
*Serratia marcescens*	4 (2.0)	1 (1.0)	1 (5.0)
*Escherichia coli*	3 (1.0)	1 (1.0)	0 (0.0)
Other	4 (2.0)	1 (1.0)	0 (0.0)
**Yeasts:**	**19 (7.0)**	**9 (12)**	**2 (10)**
*Candida*			
*albicans*	8 (3.0)	6 (8.0)	1 (5.0)
*glabrata*	3 (1.0)	2 (3.0)	1 (5.0)
*parapsilosis*	3 (1.0)	1 (1.0)	0 (0.0)
other	5 (2.0)	0 (0.0)	0 (0.0)

a Numbers are presented as number (%). Registering multiple pathogens for one CVC tip was possible.

b Coagulase negative staphylococci were type speciated using the MALDI-TOF technique.

The univariate regression analysis is described in detail in Table IV***.*** The goodness of fit in the multivariable regression analyses showed a valid chi-square value (P> 0.05) for both models. The detailed results of the multivariable regression analyses are shown in Table V***.*** In summary, 13 independent variables were selected for investigation of tip colonisation (n=257) and seven independent variables for CRI (n=74). Male gender and increased number of catheter lumens were independently associated with both catheter tip colonisation and CRI. Increased number of days with catheter and CVCs inserted in patients admitted to a medical ward were associated with increased tip colonisation, while catheters inserted in the subclavian vein were associated with decreased catheter tip colonisation compared with insertions in the jugular vein. As the frequency of CRBSI (n=20) was low, no regression analyses were performed for CRBSI. The characteristics of cases with CRBSI are presented in Table VI.

**Table IV:** Univariate analysis of potential risk factors for central venous catheter (CVC) tip colonisation and catheter-related infection (CRI). Data from between March 2019 and August 2020 in Region Skåne, Sweden^a^

Independent variables	Tip colonisation			CRI		
	Tip colonisation (n=257)	95% CI	P-value	CRI (n=74)	95% CI	P-value
Age	66 [56–75]	0.99–1.01	0.832	65 [57–75]	0.98–1.01	0.786
Sex, male	173 (67)	1.03–1.74	0.032	54 (73)	1.04–2.92	0.034
Days with catheter	8 [2–14]	1.01–1.02	0.002	6 [1–11]	0.98–1.02	0.985
Central Haemodialysis Catheter vs Central Venous Catheter	23 (9.0)	1.43–3.44	<0.001	8 (11)	1.28–5.62	0.009
Catheter lumens	-	1.41–1.72	<0.001	4 [3–5]	1.58–2.31	<0.001
**Insertion site**						
Jugular vein (reference)	236 (92)	-	-	-	-	-
Subclavian vein	17 (6.5)	0.27–0.74	0.001	5 (7.0)	0.18–1.13	0.090
Femoral vein	4 (1.5)	0.26–1.96	0.520	0 (0.0)	-	0.995
**Anticoagulant treatment before insertion**^b^	197 (77)	1b05–1.89	0.023	59 (80)	0.66–2.06	0.601
**Procoagulant treatment before insertion**	218 (85)	0.82–1.64	0.398	63 (85)	0.59–2.15	0.711
**Room intended for CVC insertion**						
Operating theatre (reference)	96 (38)	-	-	-	-	-
Intensive Care Unit	112 (44)	1.86–3.24	<0.001	37 (50)	1.91–5.35	<0.001
Room reserved for CVC-insertion	34 (13)	0.98–2.15	0.066	6 (8.0)	0.41–2.48	0.975
Patient ward	12 (5.0)	1.30–4.41	0.005	5 (7.0)	1.49–10.38	0.006
**Department of admission at CVC insertion**						
Surgical ward (reference)	83 (34)	-	-	-	-	-
Medical ward	82 (33)	1.27–2.36	<0.001	20 (27)	0.83–2.76	0.178
Intensive Care Unit	81 (33)	2.30–4.29	<0.001	27 (36)	2.11–6.46	<0.001
**High risk patient**^c^	90 (35)	1.50–2.53	<0.001	29 (39)	1.44–3.70	<0.001
**Number of skin punctures**		0.68–1.03	0.099	-	0.58–1.26	0.431
**Number of punctured blood vessels**		0.71–1.15	0.419	-	0.65–1.49	0.921
**Immediate mechanical complications**						
No (reference)	243 (95)	-	-	-	-	-
Bleeding/punctured artery	6 (2.0)	0.66–2.75	0.422	2 (3.0)	0.28–4.74	0.841
Other	8 (3.0)	0.43–2.23	0.962	2 (3.0)	0.28–4.66	0.860

a Numbers are presented as number (%) and continuous variables are presented as median [interquartile range].

b Warfarin and Non-vitamin K Antagonist Oral Anticoagulants were categorized as anticoagulative treatment, while Low Molecular Weight Heparin and Acetylsalicylic Acid were not.

c Immunocompromised patients.

**Table V:** Multivariable logistic regression analyses for tip colonisation and catheter-related infection (CRI)^a^

Independent variables	Tip colonisation			CRI		
	Odds Ratio	95% CI	P-value	Odds Ratio	95% CI	P-value
Age	1.01	0.99–1.01	0.676	1.01	0.98–1.02	0.876
Sex, male	1.50	1.11–2.04	0.008	2.06	1.13–3.78	0.019
Days with catheter	1.02	1.01–1.03	<0.001	1.02	0.99–1.04	0.130
Catheter lumens	1.57	1.37–1.80	<0.001	1.95	1.54–2.47	<0.001
**Insertion site**						
Jugular vein (reference)	-	-	-	-	-	-
Subclavian vein	0.36	0.21–0.63	<0.001	0.37	0.13–1.04	0.059
Femoral vein	0.42	0.10–1.73	0.070	-	-	0.996
**Room intended for CVC insertion**						
Operating theatre	0.68	0.45–1.03	0.071	0.62	0.30–1.27	0.190
**High risk patient**^b^	0.83	0.54–1.28	0.397	0.71	0.33–1.51	0.371
**Department of admission at CVC insertion**						
Surgical ward (reference)	-	-	-			
Medical ward	1.49	1.02–2.18	0.037			
Intensive Care Unit	1.18	0.72–1.95	0.511			
**Anticoagulant treatment before insertion**^c^	1.35	0.98–1.88	0.070			
**Immediate mechanical complications**						
No (reference)	-	-	-			
Bleeding/punctured artery	0.67	0.21–2.16	0.507			
Other	1.00	0.43–2.31	0.999			

a Abbreviations: catheter related infections (CRI), central venous catheter (CVC).

b Immunocompromised patients.

c Warfarin and NOACs were categorized as anticoagulative treatment, while Low Molecular Weight Heparin and Acetylsalicylic Acid were not.

**Table VI:** Characteristics of patients with confirmed catheter related bloodstream infection (CRBSI)^a^

Variable	No CRBSI (n=9904)	CRBSI (n=20)
Age	69 [57–76]	66 [53–71]
Sex, male	6027 (61)	11 (55)
Days with catheter	5 [2–10]	6 [1–15]
Central Haemodialysis Catheter vs Central Venous Catheter	430 (4.0)	4 (20)
**Catheter lumens**		
1	2645 (27)	2 (10)
2	2186 (22)	3 (15)
3	2793 (28)	2 (10)
4	666 (7.0)	0 (0.0)
5	994 (10)	9 (45)
**Insertion site**		
Jugular vein	8381 (85)	17 (85)
Subclavian vein	1327 (13)	3 (15)
Femoral vein	196 (2.0)	0 (0.0)
**Anticoagulant treatment at insertion**	8127 (82)	16 (80)
**Procoagulant treatment before insertion**	8577 (87)	17 (85)
**Room intended for CVC insertion**		
Operating theatre	5336 (54)	3 (15)
Intensive Care Unit	2604 (26)	12 (60)
Room reserved for CVC insertion	1321 (13)	2 (10)
Patient ward	286 (3.0)	2 (10)
**Department of admission at CVC insertion**		
Surgical ward	5042 (51)	3 (15)
Medical ward	2905 (29)	5 (25)
Intensive Care Unit	1615 (16)	9 (45)
**High risk patients**	2159 (22)	10 (50)
**Number of skin punctures**		
1	7450 (75)	18 (90)
2	1545 (16)	1 (5.0)
3	516 (5.0)	0 (0.0)
4	122 (1.0)	0 (0.0)
5 or more	60 (1.0)	0 (0.0)
**Number of punctured blood vessels**		
1	7850 (79)	18 (90)
2	1369 (14)	1 (5.0)
3	361 (4.0)	0 (0.0)
4	59 (1.0)	0 (0.0)
5	20 (0.2)	0 (0.0)
**Immediate mechanical complications**		
No	9432 (95)	20 (100)
Bleeding/punctured artery	238 (2.0)	0 (0.0)
Other	234 (2.0)	0 (0.0)

a Numbers are presented with number (%) and continuous variables are presented with median [interquartile range]. Missing data is not presented in table.

## Discussion

This observational multicentre follow-up study on 9924 CVC insertions demonstrated low incidences of CRI and CRBSI. Several associations between independent variables and CRI were identified, where catheter tips were observed as less likely to be colonised when the subclavian route was used compared to the internal jugular route and where male sex as well as increased number of catheter lumens were both associated with catheter tip colonisation and CRI. Furthermore, the automatic script-based extraction from the EHR was feasible and may be the base for future continuous CRI surveillance.

We designed the present study in an attempt to a follow-up of the results previously published by us where 1722 central venous catheter insertions inserted between the years 2011 and 2012 at a University Hospital in the same Region as the present, was investigated [4]. The previous study demonstrated an incidence of CRI and CRBSI of 1.86 and 0.62, per 1000 catheter days after the implementation of simple hygiene insertion bundles. In the present study the same point estimates were 1.2 and 0.3/1000 catheter days. These results indicate that the low incidence of CRI and CRBSI remains. However, it should be noted that there was significant time between the study periods, the present study included significantly more cases (9924 vs. 1722), used an automated script-base data-extraction from the EHR (compared to manual review), also included cases from the whole Scania Region and not only from one hospital.

Furthermore, the present study indicates that pathogens previously associated with CRI and CRBSI (Table III)*,* as presented by Thorarinsdottir *et al.* [4], still represent most cases of CVC infections at the studied hospitals. *S. epidermidis* has previously been described as the most common coagulase negative staphylococcus species in CRI [17]. In the present study, *S. epidermidis* was the only coagulase negative staphylococcus responsible for CRI and CRBSI. When comparing these results with previous national studies on CRI [7,18], we observe regional differences in pathogen growth. Hence, these findings could impact local infection management strategies to prevent certain pathogens from causing CRI. As an example, antifungal treatment could be considered when treating suspected CVC infections in regions with higher incidences of CRI caused by *Candida* spp.

In the logistic regression analysis (Table V), we identified associations between independent variables and catheter tip colonisation as well as CRI. First, longer catheterisation times were associated with catheter tip colonisation, but not with CRI. Previous studies have convincingly demonstrated that the time with the catheter correlates positively with the risk of CRI and CRBSI [7,18]. As noted by Hammarskjöld *et al.*, adequate adherence to routines advocating early removal of unnecessary catheters could minimize the effect of correlations between catheterisation time and CRI [7]. It has been suggested that catheter tip colonisation is a predisposal factor for CRI and CRBSI, hence we suggest that common practice should continue to prioritise the immediate removal of unnecessary CVCs [19]. Moreover, the current study demonstrates that CVCs inserted in the subclavian vein were associated with less catheter tip colonisation compared to insertion in the jugular vein. The insertion site, however, was not confirmed to affect the risk of CRI in this study.

Secondly, our results show that male sex and increased number of catheter lumens were associated with both catheter tip colonisation and CRI. Increased risk of catheter tip colonisation in men has previously been demonstrated in a retrospective study from 2008 by Gowardman *et al.* [16]. Studies linking gender to risk of CRI, however, are scarce. In a prospective study by Moro *et al.*, it was shown that men present an increased risk for skin colonisation at the CVC insertion site, which showed an increased risk of CRI, especially when using the jugular vein as point of insertion [20]. Furthermore, beard growth and shaving may not only facilitate pathogen multiplication, but has also been observed to reduce adherence of wound dressing materials, suggesting increased risk of bacterial contamination [21]. Increased number of CVC lumens being an associated risk factor for CRI has previously been reported [4,22], thus advocating for minimising the number of catheter lumens when choosing a CVC.

Thirdly, our results also indicate that CVCs inserted in patients admitted to a medical ward present an increased likelihood of catheter colonisation, when compared with patients admitted to a surgical ward. Several studies have evaluated the rates of CRI among inpatients receiving CVCs, with varying results [23]. As summarised by Kallen *et al.*, the differences in infection rates in different units can vary depending on the type of unit and teaching status of the facility [23]. In the light of this, it is more likely that the cause of the association between CVCs inserted in patients admitted to a medical ward and catheter tip colonisation is driven by a risk of bias in the selection of patients, where patients admitted to medical wards tend to be more immunocompromised and therefore more susceptible to infection.

By comparing this current study with previously conducted studies from the same region [4,24], it was used as a follow-up study on local CVC management. The results showed a sustained low CRI incidence. Hence, the study served as a quality improvement report indicating continuous safe CVC-routines in the studied region. Nevertheless, a low incidence is still not equal to zero and complete eradication of CRI should be the goal of any future interventions. As previously shown by Longmate *et al.* [5], rigorous hygiene and educational interventions can lead to complete elimination of CRI. Hence, a vision zero for CRI should be adopted as an ethical stance as it has been demonstrated possible to eradicate completely [25]. However, as part of an eradication process, CRI incidence should be evaluated longitudinally. Therefore, we need to find an efficient and systematic way to assess CRI over time.

In our study, as well as in more recent epidemiological studies on CRI and sepsis, but also generally, search queries and automatic script-based data extraction seems to be an efficient way of tracking medical device management and infection incidences [[8], [9], [10], [11],^26^]. As an example, Gokhale *et al.* recently presented a tool used for automatic data extraction for epidemiological research, using a process that can be verified and reproducible [26]. The study highlights how this new process of extracting available information reduces the gap between medical researchers and electronic records, enabling continuous quality surveillance.

Given that EHR (electronic health records) are increasingly used in healthcare systems for documentation, the potential power of the fully automated surveillance systems is yet to be discovered and evaluated. Data from EHRs has the potential to replace time consuming and subjective manual chart review-surveillance and may also provide continuous surveillance, such as in this study, which is the first one to our knowledge applying it to follow-up previous results. The automated electronic surveillance systems must be carefully evaluated, and the construction of the systems is resource consuming but once implemented, these systems have the potential to provide invaluable real-time quality assurance, superior to current standard. However, further research in this area, where we examine data abstraction methods across hospitals and validate automated data extraction systems, are still needed [27].

We recognise the limitations in the present study given the retrospective design. Although there is a strong tradition in the studied departments to document every CVC-insertion, it cannot be ruled out that single insertions were not documented. Although, we have tried to correct for confounders in the multivariable logistic regression analyses and the goodness of fit test was good, the presence of occult independent variables affecting the outcome still cannot be ruled out. Further, the sample size was based on the number of available insertions during the study period meaning that the power of the results is uncertain. Last, the sensitivity of the automated script-base data extraction from the EHR has not been investigated in the current study.

In summary, this large retrospective observational study demonstrated that automated data extraction of EHR data could be the base for quality assurance and epidemiological studies. Furthermore, the results indicate a sustained low incidence of CRI and CRBSI in the region and several associations between independent variables and both catheter tip colonisation and CRI, were identified. In addition, this study demonstrates that the choice of insertion site might impact the catheter tip colonisation rate. Male sex and an increased number of catheter lumens were associated with both catheter tip colonisation and CRI.

## Credit author statement

**Manuscript:** “Sustained low Catheter Relation Infection Incidence in Observational Follow-up Study of 9924 Catheters using Automatic Data Scripts as Quality Assurance for Central Venous Catheter Management” (IPIP-D-22-00062).

**Mika M. Rockholt:** Conceptualization, Methodology, Formal Analysis, Writing – Original Draft, Writing – Review & Editing, Visualization.

**Tobis Agrell:** Formal Analysis, Writing – Original Draft, Writing – Review & Editing.

**Hulda Thorarinnsdottir:** Writing – Review & Editing, Supervision.

**Thomas Kander:** Conceptualization, Methodology, Formal Analysis, Writing – Review & Editing, Supervision, Project Administration.

## Conflict of interest

Thomas Kander is on the Advisory Board of Bactiguard AB (Stockholm, Sweden) and of Anaesthesiology Intensive Therapy (Poland). The remaining authors have no conflicts of interest to declare.

## Funding

This research received funding from the following institutions: Scania Region Department of Quality Improvement (Sweden), Swedish Medical Association (Sweden), Lion's Research Fund (Sweden) and LÖF - The Swedish Patient Insurance (Sweden).
